# STC-YOLO: Small Object Detection Network for Traffic Signs in Complex Environments

**DOI:** 10.3390/s23115307

**Published:** 2023-06-03

**Authors:** Huaqing Lai, Liangyan Chen, Weihua Liu, Zi Yan, Sheng Ye

**Affiliations:** School of Electric and Electronic Engineering, Wuhan Polytechnic University, Wuhan 430023, China; lhq15527756213@163.com (H.L.); whliu2020@whpu.edu.cn (W.L.); yz9915@163.com (Z.Y.); yesheng19982023@163.com (S.Y.)

**Keywords:** small object detection, multi-scale feature fusion, loss function, data augmentation, K-means++

## Abstract

The detection of traffic signs is easily affected by changes in the weather, partial occlusion, and light intensity, which increases the number of potential safety hazards in practical applications of autonomous driving. To address this issue, a new traffic sign dataset, namely the enhanced Tsinghua-Tencent 100K (TT100K) dataset, was constructed, which includes the number of difficult samples generated using various data augmentation strategies such as fog, snow, noise, occlusion, and blur. Meanwhile, a small traffic sign detection network for complex environments based on the framework of YOLOv5 (STC-YOLO) was constructed to be suitable for complex scenes. In this network, the down-sampling multiple was adjusted, and a small object detection layer was adopted to obtain and transmit richer and more discriminative small object features. Then, a feature extraction module combining a convolutional neural network (CNN) and multi-head attention was designed to break the limitations of ordinary convolution extraction to obtain a larger receptive field. Finally, the normalized Gaussian Wasserstein distance (NWD) metric was introduced to make up for the sensitivity of the intersection over union (IoU) loss to the location deviation of tiny objects in the regression loss function. A more accurate size of the anchor boxes for small objects was achieved using the K-means++ clustering algorithm. Experiments on 45 types of sign detection results on the enhanced TT100K dataset showed that the STC-YOLO algorithm outperformed YOLOv5 by 9.3% in the mean average precision (mAP), and the performance of STC-YOLO was comparable with that of the state-of-the-art methods on the public TT100K dataset and CSUST Chinese Traffic Sign Detection Benchmark (CCTSDB2021) dataset.

## 1. Introduction

The traffic sign detection system is an important part of an intelligent transportation system. It can effectively provide the driver with current road traffic information, and it can also ensure the operational safety of the intelligent vehicle control system. In recent years, due to the far-reaching impact of this technology on traffic safety, this field has been deeply studied by many researchers.

Traditional traffic sign detection algorithms are mainly concentrated on color segmentation, combining features such as the shape and contour for feature extraction, and then realizing the recognition of traffic sign by completing feature classification through classifiers [1,2,3,4,5,6]. The handmade features in traditional techniques are human exhaustion and a lack of sufficient robustness to deal with complex and changeable traffic environments. In recent years, traffic sign detection algorithms based on deep convolutional neural networks have been widely developed. They are mainly divided into two categories: the two-stage object detection algorithm represented by the region-based convolutional network (R-CNN) series [7,8,9], and the one-stage object detection algorithm represented by the you only look once (YOLO) series [10,11,12] and the single shot multibox detector (SSD) series [13,14]. The two-stage algorithm has achieved remarkable results in accuracy, but the lack of real-time performance means that it is difficult to apply most of the methods to practical detection tasks. Researchers are more concerned with the one-stage algorithm because it can predict the object categories and generate the bounding boxes simultaneously, being competent for detection tasks with high real-time requirements. Zhang et al. [15] introduced a multi-scale spatial pyramid pooling block based on the YOLOv3 [10] algorithm, aiming to accurately realize the real-time location and classification of traffic signs. The mean average precision (mAP) of the algorithm on the Tsinghua-Tencent 100K (TT100K) dataset [16] was satisfactory, but it detected only 23.81 frames per second (FPS). Wu et al. [17] proposed a traffic sign detection model based on SSD [13] combined with a receptive field module (RFM) and path aggregation network (PAN) [18], which achieved a 95.4% and 95.9% mAP on the German Traffic Sign Detection Benchmark (GTSDB) dataset [19] and CSUST Chinese Traffic Sign Detection Benchmark (CCTSDB) dataset [20], respectively, but it has high requirements for the storage capacity and computing power of the device. Yan et al. [21] proposed an auxiliary information enhanced YOLO algorithm based on YOLOv5, which achieved a detection speed of 84.8% mAP and 100.7 FPS on the TT100K dataset, but its robustness against complex scenes such as extreme weather and lighting changes has not been verified.

The research on the detection of traffic signs in harsh environments such as fog, strong light, and insufficient light has attracted the attention of many scholars. Hnewa et al. [22] proposed a novel multi-scale domain adaptive YOLO framework, which extracts domain-invariant features from blurred long-distance image regions and has a significant effect on foggy image datasets. Fan et al. [23] proposed a multi-scale traffic sign detection algorithm based on an attention mechanism, which can effectively reduce the effect of illumination changes on traffic sign detection. Zhou et al. [24] proposed an attention network based on high-resolution traffic sign classification to overcome the complex factors of icy and snowy environments. However, the above methods used a single scene and cannot be effectively applied to multi-scene detection tasks.

The public CCTSDB2021 and TT100K datasets are the datasets most widely used to evaluate the performance of traffic sign detection, and small objects account for 44% and 84% of the datasets, respectively, according to the definition of a small object by the International Society of Optics and Photonics (SPIE) [25]. Additionally, pictures in these datasets mainly involve daytime and normal weather scenarios. Methods and datasets that focus on traffic sign multi-scene detection images are scarce. In this work, the enhanced TT100K dataset was constructed, through a variety of data augmentation techniques, such as fog, snow, and lighting changes. Additionally, inspired by the state-of-the-art YOLO series of object detection algorithms, a small traffic sign detection network for complex environment (STC-YOLO), is proposed, which outperforms all the state-of-the-art detection algorithms. The source code used in this study are available at https://github.com/987654321-lhq/STC-YOLO (accessed on 28 May 2023).

The main contributions of this paper are outlined as follows:

(1) The down-sampling multiple was adjusted, and a small object detection layer was added to reduce the loss of small object information transmission during the down-sampling operation.
(2) The Swin Transformer structure was combined with a convolutional neural network (CNN) for local relevance as well as global modelling capabilities.
(3) Complete-IoU (CIoU) and the normalized Gaussian Wasserstein distance (NWD) metric were combined as loss functions, and the robustness of the model in small object detection was improved by adjusting the proportional relationship.
(4) The K-means++ algorithm was used to obtain a new initialized anchor box size by clustering and analyzing the instance label information, which can improve the matching degree between the anchor boxes and the real samples.

The rest of this paper is structured as follows: Section 2 introduces the relevant research on small object detection and traffic sign detection. Section 3 describes the proposed methods in detail. Section 4 describes the experimental results and analysis, including comparative studies and ablation studies. Finally, Section 5 presents the discussion of the experimental results, and Section 6 includes the conclusion and future prospects of this paper.

## 2. Related Works

### 2.1. Small Object Detection

There are usually two ways to define small objects. One definition states that the object size must be smaller than 0.12% of the original size to be regarded as a small object. This paper takes this as a reference. The other is an absolute size definition, that is, the object size must be smaller than 32 × 32 pixels. Therefore, small object detection has always been a difficult topic to address in the field of object detection. At present, multi-scale fusion, the receptive field angle, high-resolution detection, and context-aware detection are the main approaches to small object detection. In high-resolution detection [26,27], high-resolution feature maps are established and predicted to obtain fine details, but context information is lost. In addition, to obtain the context information of the object, there are several methods [28,29] that use the top-down and bottom-up paths to fuse the features of different layers, which can greatly increase their receptive field. In this paper, the feature pyramid network (FPN) [30] + PAN was used as the feature fusion module of the network, and a multiple attention mechanism was introduced in the model backbone to enhance the learning of context and expand the receptive field, so as to effectively improve the accuracy of small object detection.

### 2.2. Traffic Signs Detection

The key to traffic sign detection is to extract distinguishable features. Due to limitations in the computer power and available dataset size, the performance of traditional methods depends on the effectiveness of the manual extraction of features, such as color-based [31,32] and shape-based methods [33,34]. These methods are also easily affected by factors such as extreme weather, illumination changes, variable shooting angles, and obstacles, and can only be applied to limited scenes.

In order to promote traffic sign detection in real scenes, many authors have published excellent traffic sign datasets, such as the Laboratory for Intelligent and Safe Automobiles (LISA) dataset [35], GTSDB, CCTSDB, and TT100K. Since the TT100K dataset covers partial occlusion, illumination changes, and viewing angle changes, it is closer to the real scene than other datasets. With the development of deep learning technology, and the publication of several excellent public datasets, the performance of traffic sign detection algorithms based on deep learning has been significantly improved compared with the traditional traffic sign detection algorithms. Zhang et al. [36] used the Cascade R-CNN [8] combined with the sample balance method to detect traffic signs, achieving ideal detection results on both CCTSDB and GTSDB. Sun et al. [37] proposed a feature expression enhanced SSD detection algorithm, which achieved an 81.26% and 90.52% mAP on TT100K and CCTSDB, respectively. However, the detection speed of this algorithm was only 22.86 FPS and 25.08 FPS, which could not achieve real-time performance. Liu et al. [38] proposed a symmetric traffic sign detection algorithm, which optimizes the delay problem by reducing the computing overhead of the network and, at the same time, improves the traffic sign detection performance in complex environments, such as scale and illumination changes, achieving a 97.8% mAP and 84 FPS on the CCTSDB dataset. However, the integration of multiple modules leads to insufficient global information acquisition.

## 3. Materials and Methods

**Figure 1:**
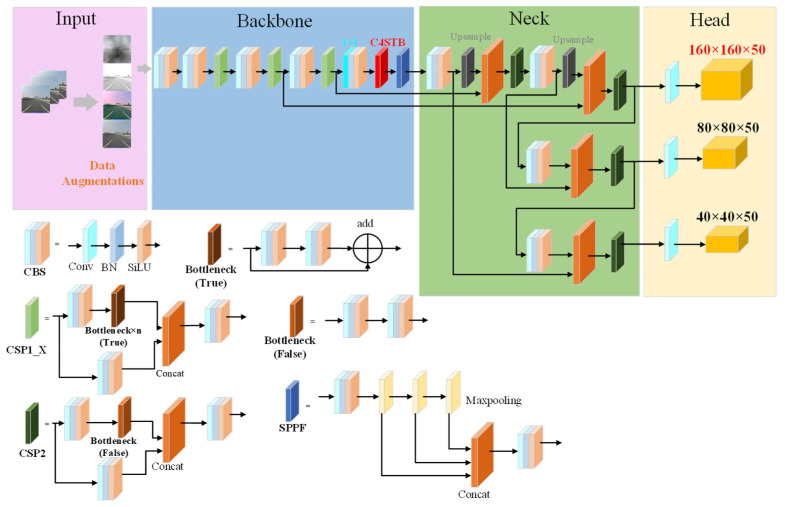
The overall structure of the small traffic sign detection network for complex environment (STC-YOLO).

The YOLOv5 model in the YOLO series has many advantages such as high detection accuracy, fast operation speed, and easy deployment, and has been widely used in many industrial fields. However, due to its poor detection performance on small objects, the network needs to be improved to improve its performance in small object detection. Considering the high requirements of traffic sign detection for model accuracy and speed, YOLOv5 was selected as the baseline network for subsequent improvement in this paper, and a small traffic sign detection network, STC-YOLO, was constructed for complex scenes. The overall structure is shown in Figure 1. In the feature extraction part, a 16 times down-sampling operation was applied instead of 32 times down-sampling, and shallow branching was added to reduce the loss of small object information during feature propagation. A feature extraction module with a stronger characterization ability was designed to replace the C3 module to make up for the decrease in the receptive field caused by the adjustment of the subsampling operation. On the basis of CIoU loss, the NWD was introduced to calculate the localization loss to balance the sensitivity of the IoU to the location deviation of tiny objects. The K-means++ algorithm was used to replace the K-means algorithm in the original network to improve the matching degree of small objects.

### 3.1. Feature Pyramid

**Figure 2:**
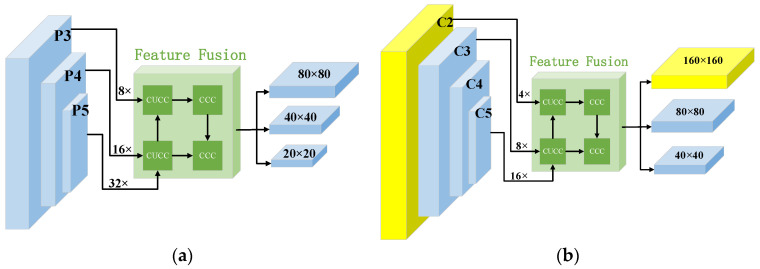
Structures of the feature pyramids: (**a**) the path aggregation network (PANet) structure; (**b**) the multiscale path aggregation network (MPANet) structure.

The neck part of the YOLOv5 model uses the information obtained from the backbone part to strengthen the representation capability of features through the FPN and PAN structure. The structure is shown in Figure 2a, where CUCC represents the Conv, Upsample, Concat and C3 modules, respectively, while CCC represents the Conv, Concat, and C3 modules, respectively. The backbone network is used to extract features from the object image and output three feature maps of different scales. {P3, P4, P5} are the feature maps after the input image has been down-sampled {8, 16, 32} times, respectively. In the feature extraction pyramid, the receptive field of the 32 times down-sampling is the largest, and the area of the mapped full-size image is the largest, which is more suitable for predicting large objects. However, most objects in traffic sign images are small objects composed of dozens of or even a few pixels, and effective feature information (i.e., color, shape, size, and texture) is scarce, which leads to poor detection effects for small objects. In order to ensure the transmission of more small object details and output feature maps with a stronger characterization capability for small objects, this paper improved the multi-scale structure on the basis of the original feature pyramid structure. The multiscale path aggregation network (MPANet) structure is shown in Figure 2b.

The small-scale prediction output of the YOLOv5 model is a feature map with a size of 20 × 20. An image with a size of 2048 × 2048 in the TT100K dataset is taken as an example. When the image is down-sampled to 20 × 20, objects with a size smaller than 103 × 103 are compressed to less than one pixel; most of the traffic sign objects in the image are smaller than 103×103, meaning the small-scale prediction in YOLOv5 is of little significance for small object detection. Directly cutting the small-scale detection layer will cause a lack of semantic information among the deep networks, which in turn will affect the accuracy of the fine-grained classification. {C2, C3, C4, C5} are the feature maps after the input image has been down-sampled {4, 8, 16, 16} times, respectively. In this work, the 32 times down-sampling layer was replaced with a 16 times convolutional layer in the backbone network, and at the 16th layer, the feature map output after 4 times down-sampling by the backbone part and the feature map of the neck part after 2 times up-sampling processing were bonded in parallel to obtain a 160 × 160-size feature map for predicting small objects. This feature map has a smaller receptive field and rich object information. After multi-scale fusion, it can better learn object features, enhance the capture ability of the network for smaller objects, and improve the object detection effect.

### 3.2. C4STB Module

**Figure 3:**
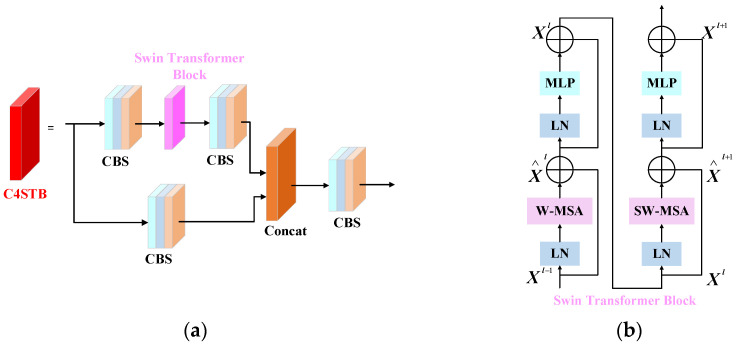
(**a**) Structure of the four convolutional and Swin Transformer Block (C4STB) module; (**b**) structure of the Swin Transformer Block (STB).

Aiming at the problem that traffic signs are not easy to detect due to their small sizes, leading to a poor detection performance, the above method uses 1 × 1 convolution instead of a down-sampling 3 × 3 convolution operation to ensure the transmission of more detailed information. Although CNNs have achieved great success in image processing, their limited perceptual range limits their ability to capture global contextual information. In contrast, the Swin Transformer [39] adopts a more flexible self-attention mechanism, which can better communicate global semantic information, and is outstanding in extracting global semantic information and achieving the best performance. On this basis, a feature extraction module with a stronger characterization ability was constructed by combining four convolutional modules and the Swin Transformer Block (C4STB). Its structure is shown in Figure 3a. The bottleneck in the YOLOv5 feature extraction unit was replaced with the Swin Transformer Block (STB) in the Swin Transformer, the receptive field was expanded with the help of a window self-attention module, and 3 × 3 convolution was added to enhance the local information of the object.

As shown in Figure 3b, the STB structure consists of windows multi-head self-attention (W-MSA), shifted windows multi-head self-attention (SW-MSA), and multi-layer perceptron (MLP). A residual connection is applied after each MSA and MLP, and a layer norm (LN) layer is inserted between the modules. This part can be expressed as follows:

(1) $${\hat{X}}^{l}=W-MSA(LN(X^{l-1})+X^{l-1})$$

(2) $$X^{l}=MLP(LN({\hat{X}}^{l})+{\hat{X}}^{l})$$

(3) $${\hat{X}}^{l+1}=SW-MSA(LN(X^{l})+X^{l})$$

(4) $$X^{l+1}=MLP(LN({\hat{X}}^{l+1})+{\hat{X}}^{l+1})$$

where ${\hat{X}}^{l}$ and $X^{l}$ denote the output features of the W-MSA (SW-MSA) module and the MLP module for block l, respectively.

**Figure 4:**
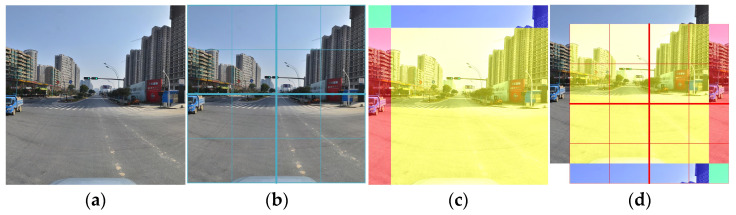
The mechanism of action of the shifted windows. (**a**) the input images; (**b**) window segmentation of the input image through windows multi-head self-attention (W-MSA); (**c**) action of the shifted windows; (**d**) a different window segmentation method using shifted windows multi-head self-attention (SW-MSA).

In the traditional ViT, multi-head self-attention (MSA) needs to process all of the image information at the same time, so the computational complexity is relatively high. In contrast, W-MSA in the STB uses a window as a unit (the window size is set to 7 by default) to control the calculation area with less computation. This method reduces the network complexity such that the computational complexity scales linearly with the image size. However, this method will also block the information transmission between different windows, making it necessary to use the SW-MSA module to solve this problem, effectively extract the distance information, and achieve a more accurate semantic understanding. As shown in Figure 4, compared with W-MSA, SW-MSA adds a shift operation and establishes the information interaction between different windows without increasing the computational overhead. The W-MSA adopts a regular window-partitioning mechanism for the input images and calculates the self-attention within each window. The result of window segmentation is shown in Figure 4b. The SW-MSA module will shift when performing window division, thus generating new windows. With half of the window size as the step size, each period of the image is moved to the upper left direction, and then the blue and red areas in Figure 4c move to the lower and right sides of the image, respectively, finally achieving the effect after the window division offset, as shown in Figure 4d.

### 3.3. Loss Function

The original YOLOv5 network uses CIoU [40] as the regression loss function. The CIoU function considers three important geometric metrics, which are the distance of the center point, overlap area, and aspect ratio. Its performance is better than that of other methods and can provide the movement direction when the bounding boxes do not overlap. The calculation formula of CIoU is as follows:

(5) $$CIoU=1-IoU+\frac{\rho^{2}(b_{A},b_{B})}{c^{2}}+\alpha v$$

(6) $$a=\frac{v}{(1-IoU)+v}$$

(7) $$v=\frac{4}{\pi^{2}}{\left(arctan\frac{w_{B}}{h_{B}}-arctan\frac{w_{A}}{h_{A}}\right)}^{2}$$

where $\rho^{2}(b_{A},b_{B})$ indicates the Euclidean distance between the center points of the predicted and real boxes, and c represents the diagonal distance of the smallest circumscribed rectangle of the two boxes; the weight factor is denoted by α, and the aspect ratio consistency is denoted by v; $w_{A}$ and $h_{A}$ represent the width and height of the predicted box; $w_{B}$ and $h_{B}$ represent the width and height of the real box.

Considering that intersection over union (IoU)-based metrics (such as the IoU itself and its extensions) [41,42] are excessively sensitive to the location deviation of the tiny objects, applying anchor-based detectors results in a drastic deterioration of the detection performance. To alleviate this, this paper combines CIoU and NWD [43] to calculate the localization loss. The bounding boxes are first modeled as a two-dimensional Gaussian distribution, and the similarity between them is calculated using the Gaussian distribution corresponding to the predicted object and the real object. Next, the normalized Wasserstein distance between them is calculated according to Equation (9). Finally, the localization loss is calculated according to the proportional relationship between CIoU and NWD in Equation (10), which is defined as follows:

(8) $$W_{2}^{2}(N_{A},N_{B})=||([cx_{A},cy_{B},\frac{w_{A}}{2},\frac{h_{A}}{2}{]}^{T},[cx_{B},cy_{B},\frac{w_{B}}{2},\frac{h_{B}}{2}{]}^{T})|{|}_{2}^{2}$$

(9) $$NWD(N_{A},N_{B})=exp(-\frac{\sqrt{w_{2}^{2}(N_{A},N_{B})}}{C})$$

(10) $$L_{loc}=(1-\beta )*(1-NWD(N_{A},N_{B}))+\beta *(1-CIoU)$$

where $N_{A}$ and $N_{B}$ are Gaussian distributions modeled by $A=(cx_{A},cy_{A},w_{A},h_{A})$ and $B=(cx_{B},cy_{B},w_{B},h_{B})$, $W_{2}^{2}(N_{A},N_{B})$ is a distance measure, c is a constant closely related to the dataset, and β is the weight proportional coefficient.

For the detected objects, regardless of whether the objects overlap or not, localization loss can be measured using the distribution similarity. In addition, NWD is not sensitive to the scale of the objects, meaning it is more suitable for measuring the similarity between small objects [44]. In the regression loss function, NWD loss is added to make up for the disadvantage of CIoU loss in small object detection, and CIoU loss is retained, which makes the algorithm converge faster when predicting the bounding box localization and improves the model performance.

### 3.4. Anchor Box

The original YOLOv5s model uses the K-means algorithm to cluster the COCO dataset [45], so that the feature maps with different sizes have three anchor boxes with different fixed widths and heights. However, the K-means algorithm is affected by the random selection of the initial cluster center, which may cause the initial cluster center to be far away from the optimal cluster center location; this not only affects the convergence speed of the model, but also leads to poor detection results. At the same time, considering that the number of large and medium objects in the COCO dataset accounts for the majority, the size of the generated anchors is too large to meet the actual needs of traffic sign detection. In order to solve the above problems, this paper uses the K-means++ algorithm to re-cluster all labeled object frames in the training dataset.

The K-means++ clustering algorithm is an optimization algorithm based on the K-means algorithm. Its main purpose is to improve the selection of the initial points, make the anchor box size of the training dataset more appropriate, and improve the detection accuracy of the model for small objects. The algorithm selects a random sample point from the dataset as the first initialized cluster center. Then, for each sample point, the shortest distance between it and the current clustering center is calculated, and the probability of each point becoming the next clustering center is calculated using the distance information. Following this, the point with the largest probability value is selected from these probability values as the next clustering center, and the above calculation steps are repeated until k clustering centers are selected. Finally, for each sample in the dataset, its distance to the k clustering centers is calculated and assigned to the corresponding class of the clustering center with the smallest distance. The cluster center is updated iteratively until the position of the cluster center no longer changes. In this way, the K-means++ clustering algorithm can better adjust the position of the cluster center, so as to obtain more accurate classification results.

## 4. Experimental Results and Analysis

### 4.1. Experimental Dataset

#### 4.1.1. Dataset

**Figure 5:**
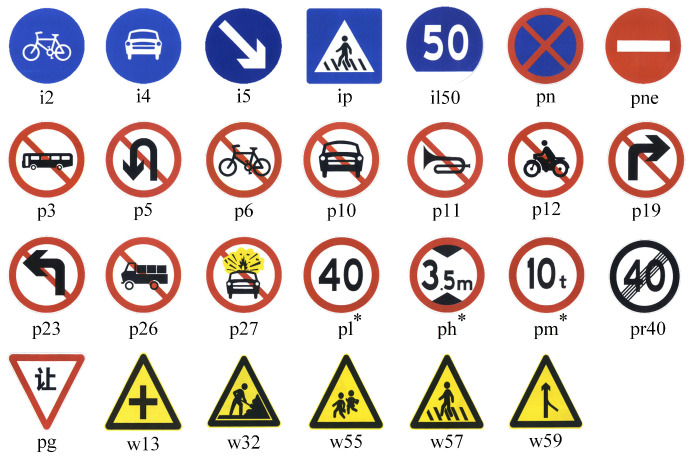
Traffic sign categories in Tsinghua-Tencent 100K (TT100K).

The TT100K traffic sign dataset provides 100,000 high-resolution images (with a resolution of 2048 × 2048), containing 30,000 traffic sign instances, and the size distribution of the instances in the images ranges from 16 × 20 to 160 × 160 pixels. A total of 45 categories with more than 50 instances were chosen in this experiment. The dataset has a total of 7962 images containing complete annotation information, of which 6262 were selected as the training set and 1700 were selected as the test set. The corresponding flag images and category names are shown in Figure 5, where ${pl}^{*}$ includes pl100, pl120, pl20, pl30, pl40, pl5, pl50, pl60, pl70, and pl80; ${pm}^{*}$ includes pm20, pm30, and pm55; ${ph}^{*}$ includes ph4, ph4.5, and ph5; and ${il}^{*}$ includes il100, il60, and il80.

The CCTSDB2021 dataset contains 17,856 images, with 15,886 images for training and 1970 images for testing. It contains scenes such as urban roads and highways, with resolutions between 600 × 900 and 1024 × 768. The size distribution of the traffic signs in the images ranges from 20 × 20 to 573 × 557 pixels. There are three main categories of traffic signs, namely “warning”, “prohibited”, and “mandatory”.

#### 4.1.2. Data Augmentation

**Figure 6:**
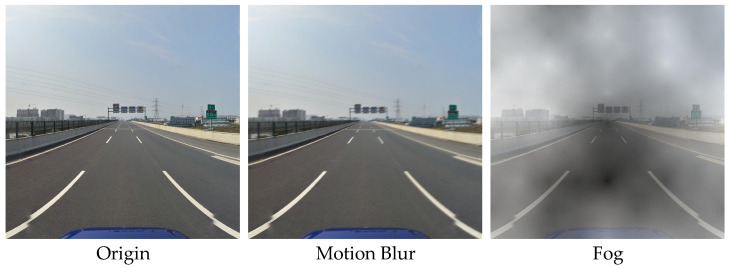

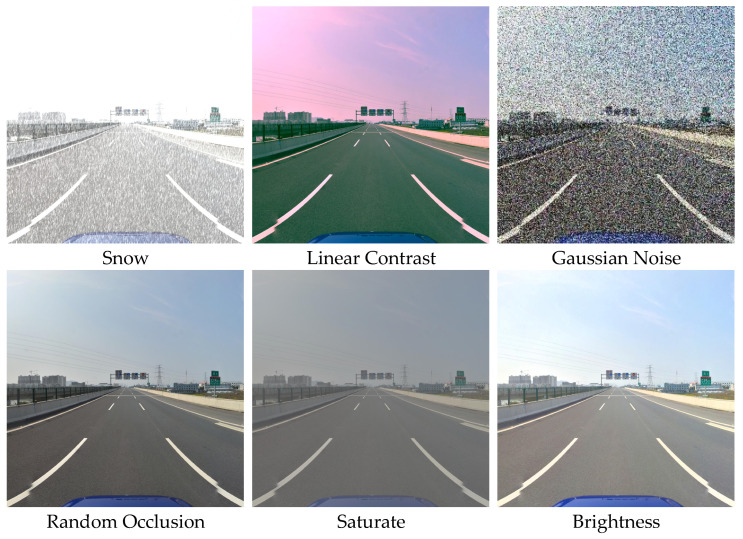
Use of data enhancement methods to generate images of a randomly selected image from the TT100K dataset.

In the face of the complex environments in traffic sign detection, this paper followed the approach of enhancing corruption proposed in the literature [36] and chose to enhance the TT100K training dataset. Considering the environmental conditions and the size of the traffic signs dataset, we added fog, snow, Gaussian noise, random occlusion, and motion blur and adjusted the contrast, brightness, and saturation of the randomly selected image to expand the minority samples. After data augmentation, the TT100K dataset was extended to 22,776 images as the enhanced TT100K dataset, of which 21,076 images (14,814 in harsh environments and 6262 in natural environments) were selected as the training set and 1700 images were selected as the test set. The enhancement results of one picture in the TT100K dataset are shown in Figure 6.

### 4.2. Experimental Environment and Parameter Settings

In this work, all experiments were conducted using the Windows11 operating system, 64 GB RAM, and a GTX-3090 graphics card with 24 GB of video memory; the deep learning framework used was Pytorch1.11.0; and the programming language was Python 3.8.

The optimization algorithm used for model training was stochastic gradient descent (SGD). The initial learning rate was 0.01, the momentum was 0.937, and the weight decay coefficient was 0.0005. In addition, the model was trained for 200 epochs, the batch size of the TT100K dataset was set to 32, and the batch size of the CCTSDB2021 dataset was set to 16.

### 4.3. Experimental Evaluation Index

The evaluation indexes are mainly divided into two aspects: detection accuracy and detection speed. Precision (P) mainly measures the degree of model error detection; recall (R) mainly measures the degree of model missed detection; average precision (AP) is the area under the P–R curve; mAP is the AP average of all categories. They are calculated as follows:

(11) $$P=\frac{TP}{TP+FP}$$

(12) $$R=\frac{TP}{TP+FN}$$

(13) $$AP=\int_{0}^{1}P(R)dR$$

(14) $$mAP=\frac{1}{n}\sum_{j=1}^{n}AP(j)$$

where TP means true positive, TN means true negative, FP means false positive, and FN means false negative. n is the number of categories; AP(j) represents the AP of the jth category.

The detection speed adopts the FPS, which represents the number of images that can be processed per second.

The model complexity uses parameters, and the specific calculation formula is as follows:

(15) $$Params=C_{o}\times (k_{w}\times k_{h}\times C_{i}+1)$$

where $C_{o}$ represents the number of output channels, $C_{i}$ represents the number of input channels, and $k_{w}$ and $k_{h}$ represent the width and height of the convolution kernel, respectively.

### 4.4. Experimental Analysis

#### 4.4.1. Feature Fusion Layer Improvement Experiment

To determine the final number of detection heads of the model, different detection head branches and their performance were compared. The experimental results are shown in Table 1. As can be seen, the network model with the p2 detection head outperformed the mAP of YOLOv5s by about 0.4% to 6.8%. With a reduction in the down-sampling multiple and the addition of the p2 detection head module, the mAP showed the largest increase and the lowest parameter number compared with YOLOv5s, which proves that this structure transmits more small object information. Considering the number of parameters and the detection accuracy comprehensively, the prediction branch corresponding to the improved {p2, p3, p4} was selected as the output detection head.

#### 4.4.2. Ablation Study

To verify the contribution of each module to the model performance, the added modules and YOLOv5s were combined to conduct ablation experiments on the enhanced TT100K dataset. The experimental results are shown in Table 2. Table 2 lists the four evaluation indicators: AP, AR, mAP, and FPS. Compared with YOLOv5s, the model proposed in this paper increased the AP value by 7.9%, the AR value by 9.7%, and the mAP value by 9.3%, and the speed was only slightly decreased (from 101.01 FPS to 87.71 FPS). This shows that the network model proposed in this paper can substantially improve the detection accuracy of small objects on the basis of ensuring real-time performance.

Each module adopted in this work improved the detection accuracy of the network to some extent. Compared with the YOLOv5s, the MPANet module brought significant improvements (mAP from 79.6% to 86.4%), with the AP, AR, and mAP being improved by 5.4%, 6.0%, and 6.8%, respectively. This shows that the prediction of larger sizes can make better use of the detailed information of traffic signs in the image, so as to detect small traffic signs more accurately. When the C4STB module proposed in this paper was applied to the YOLOv5s model, the AP, AR, and mAP were increased by 1.8%, 1.1%, and 1.1%, respectively. This shows that the C4STB module can effectively extract features with a better discriminating ability for small object detection and, at the same time, expand the receptive field to ensure the accuracy of medium and large-size objects. When the combination of NWD and CIoU was used as the loss function, the AP, AR, and mAP were increased by 5.7%, 2.8%, and 2.5%, respectively. This shows that the introduction of NWD into the regression loss function is helpful in improving the sensitivity of the IoU-based metrics to small object position deviations, thereby improving the detection accuracy of small objects. The K-means++ clustering algorithm was used to obtain a set of anchor boxes that correspond to the traffic sign dataset, which increased the AP, AR, and mAP by 5.4%, 3.7%, and 2.1%, respectively. This shows that generating candidate boxes that are more suitable for small object detection can better cover small objects in the dataset and effectively solve the problem of the low detection rate of candidate boxes in small object datasets.

**Figure 7:**
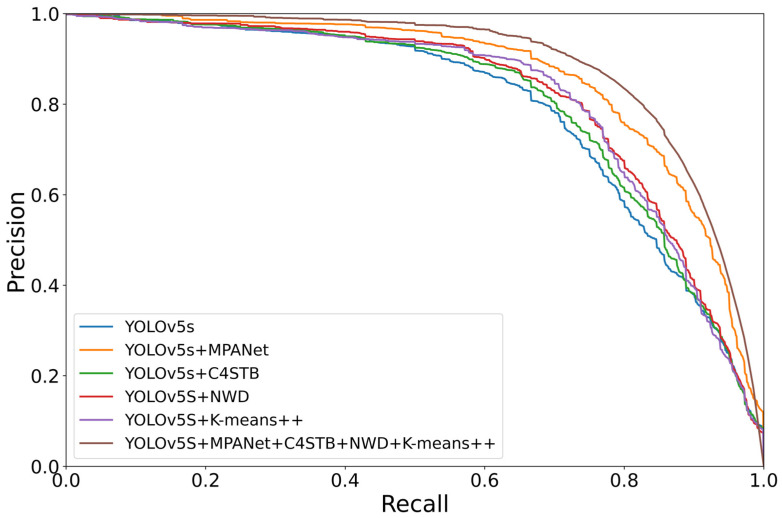
Comparison of P-R curves of each module in the STC-YOLO network.

The P–R curve with each improved module added to the YOLOv5s network was drawn under the same axis, as shown in Figure 7. It can be clearly seen that the curve with the MPANet, C4STB, NWD, and K-means ++ modules added at the same time covers the curve with a single module added. Intuitively, it is concluded that each improved module in this paper provides a certain performance improvement to the network.

### 4.5. Comparison Experiment

#### 4.5.1. Performance on the Enhanced TT100K Dataset

To confirm the validity of the network model proposed in this work, the detection results of three mainstream object detection networks, namely YOLOv3, YOLOv6, and YOLOv7, were reproduced on the enhanced TT100K dataset, and compared with those of STC-YOLO and YOLOv5s. The comparison results are shown in Table 3. It can be seen that the model proposed in this paper achieved relatively excellent results in the AP, AR, mAP, and FPS. The mAP of STC-YOLO was 88.9%, which was 5.8%, 7.6%, and 31.8% higher than that of YOLOv3, YOLOv6, and YOLOv7, respectively. The detailed mAP values for each algorithm in each category are shown in Table 4. It can be seen that the STC-YOLO model achieved the best performance in most of the 45 categories.

**Figure 8:**
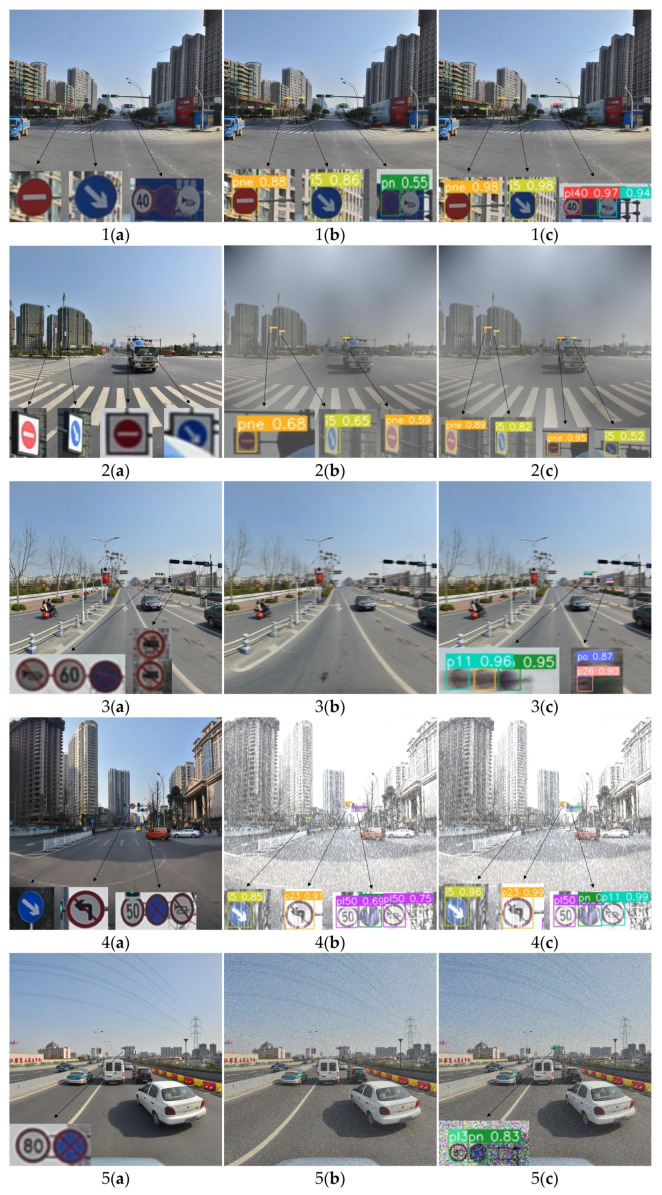

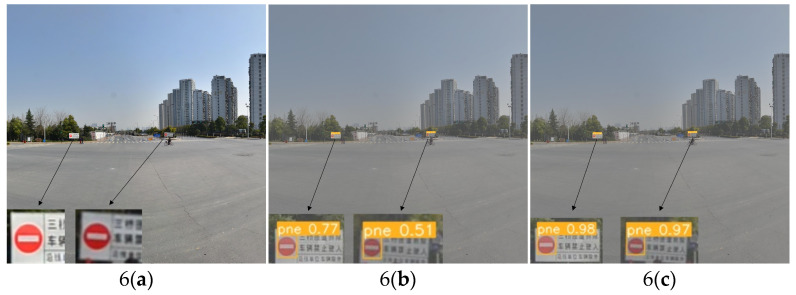
Detection examples from the enhanced TT100K testing set with a variety of conditions (1: normal; 2: foggy; 3: motion blur; 4: snowy; 5: noisy; 6: lighting changes). (**a**) The original image. (**b**) The image detected by the YOLOv5s network. (**c**) The image detected by the STC-YOLO network. All of the object area is enlarged and displayed.

The visualization of traffic sign detection using STC-YOLO and the YOLOv5s network on the enhanced TT100K dataset is presented in Figure 8. The tested images were processed by simulating noise, blur, weather and lighting, and all images used for testing were unused images during training.

In Figure 8(1b,1c), it can be seen that under the normal condition, the proposed method accurately detected each traffic sign, while YOLOv5s showed missed detection in the detection of the small objects “pl40” and “p11”. This type of missed detection was also reflected in the foggy conditions, as presented Figure 8(2b). The decrease in image clarity leads to increasing difficulty in detecting traffic signs; YOLOv5s missed a small object from the “i5” category, while STC-YOLO realized the correct detection of all signs. After adding motion blur, which caused interference in the image, the YOLOv5 network did not detect any objects, while STC-YOLO accurately detected all the objects in the image, as presented in Figure 8(3b,3c). Under the interference of rain and snow, YOLOv5 showed false detection in the detection of the “p11” sign, while STC-YOLO correctly detected all the objects in the image, as shows in Figure 8(4b,4c). Comparing Figure 8(5b,5c), it can be found that under the interference of noise, YOLOv5 missed the small objects “pl30” and “pn” in the distance, while STC-YOLO correctly detected all the signs in the image. When the image was disturbed by illumination changes, both YOLOv5 and STC-YOLO correctly detected the traffic signs, but STC-YOLO had a higher detection accuracy for the “pne” category of small objects, as shown in Figure 8(6b,6c).

#### 4.5.2. Performance on the TT100K Dataset

To further prove the superiority of the network model proposed in this paper, the STC-YOLO network was compared with five other networks, namely SSD + Aligned Matching [46], TSR-SA [47], PSG-Yolov5 [48], AIE-YOLO [21], and YOLOv5s, on the TT100K dataset. These networks are all common frameworks and represent the latest improvements in the field of small object detection in recent years. The experimental results are shown in Table 5. It can be seen that, compared with YOLOv5s, the mAP of the network proposed in this paper increased by 12.9% in real-time detection. Compared with the other state-of-the-art methods, the proposed network also showed certain advantages in detection accuracy and real detection, with 88.49 FPS on GPU 3090. Overall, the network proposed in this paper takes into account both the accuracy and real-time performance and has a better performance in detecting small traffic signs.

#### 4.5.3. Performance on the CCTSDB2021 Dataset

To ensure the authenticity and effectiveness of the proposed network, experiments on the CCTSDB2021 dataset were conducted. The comparison of STC-YOLO with state-of-the-art methods on the CCTSDB2021 dataset is shown in Table 6. It can be seen that STC-YOLO outperformed the one-stage methods ESSD [37], YOLOv3 + MAF + SIA [49], M-YOLO [38], and YOLOv5s in the mAP. Compared with the two-stage method Faster R-CNN + ACFPN + Auto Augment [50], STC-YOLO achieved a comparable mAP and outstanding speed for real detection.

The effectiveness of the proposed STC-YOLO model in small object detection was also verified on the Visdrone2019 public dataset [51], in which there are 288 video clips with a total of 10,209 still images captured by various drone cameras and 10 categories of objects. The detailed experimental results can be found in the Supplementary Materials.

## 5. Discussion

The novelty of this study lies in the amplification of a large amount of new data related to traffic signs. These data cover partial occlusion, illumination changes, view changes, and extreme weather conditions. In addition, the robustness of the model proposed in this paper to complex environments and small objects was tested on multiple publicly available datasets such as TT100K and CCTSDB2021.

It can be seen from the results in Section 4.4 that by reducing the subsampling multiple and designing a larger prediction head, the model proposed in this paper performed better in small object detection and obtained the highest mAP. Even if the detection speed decreases slightly, it can still meet the real-time requirements. The contribution of the module additions to ablation was also discussed. As shown in Table 2, the addition of each module improves the network detection accuracy to a certain extent. The MPANet module aims to feed back more shallow features after multi-scale fusion, so as to enhance the ability of the network to capture smaller objects. The C4STB module adopts the design of the Swin Transformer structure combined with a convolutional neural network to make up for the problem of the reduced receptive field caused by the reduced subsampling operation, and also to solve the limitation of the global context information acquisition ability caused by the reduced receptive field. The introduction of the NWD index combined with the CIoU loss function as an index to measure the similarity between the real boxes and the predicted boxes, which can capture the spatial information of the object better, reduces the impact of the change in the size or shape of the object on the detection results, thus significantly improving the accuracy of object detection. The K-means++ algorithm is used to cluster the label boxes and optimize the initial centroid, avoiding the situation where the k-means algorithm may fall into the local optimal solution.

Based on the comparative experiments in Section 4.5, the STC-YOLO network showed higher accuracy and computational efficiency compared to other mainstream networks. In addition, the model’s performance was also improved under interference such as snow, fog, noise, motion blur, and partial occlusion. However, there are still some limitations. First, this study did not take into account all natural conditions, such as nighttime conditions and when traffic signs are faded or damaged. Secondly, because the fusion of multiple modules may increase the amount of computation, the detection speed is slightly decreased. In future studies, it is planned to optimize the STC-YOLO model for more complex environments.

## 6. Conclusions

To deal with traffic sign detection in complex real environments, the TT100K public dataset was expanded to 22,776 images, and a traffic sign detection network, STC-YOLO, based on the framework of YOLOv5 was constructed. The STC-YOLO network achieved 88.9% in the mAP, which was 9.3% higher than that of the original YOLOv5s. In the STC-YOLO network, aiming at the small size and difficult localization of traffic signs, the ability to capture smaller objects was enhanced by adjusting the down-sampling multiple. The NWD metric was introduced to make up for the sensitivity of IoU loss to the positional deviation of tiny objects. The K-means++ clustering algorithm was used to obtain the anchor box scales that are more suitable for the traffic sign dataset. Additionally in the feature fusion stage, the feature enhancement module C4STB was designed to take the local and global information obtained from the feature map as the low-level feature of the fusion. In addition, the adaptability experiment on the TT100K and CCTSDB2021 datasets further proved the STC-YOLO network’s superiority. The STC-YOLO network is more suitable for small object detection tasks in complex environments. This work provides ideas for environment perception in autonomous driving and can be extended to the field of small object detection. However, there are many difficulties in detecting traffic signs at night, such as street lighting and reflected light interference. The robustness of the STC-YOLO algorithm to nighttime conditions has not been verified. In the future, datasets could be collected to focus on traffic sign detection in night scenes. Finally, further optimization of the performance and stability of the model should be carried out, and the design and development of mobile terminal systems should be focused on.

## Figures and Tables

**Table 1:** Number of detection head branches and their performance.

Detection Head	mAP (%)	Params (M)	Speed (FPS)
{p3, p4, p5}	79.6	7.14	101.01
{p2, p3, p4}	80	7.17	90.90
{p2, p3, p4, p5}	86.1	7.87	83.33
Improved {p2, p3, p4}	86.4	6.05	82.64

**Table 2:** Effects of each component in this method. The results on the enhanced TT100K dataset are reported.

Baseline	MPANet	C4STB	NWD&CIoU	K-Means++	AP (%)	AR (%)	mAP (%)	Speed (FPS)
√					80.7	73.5	79.6	101.01
√	√				86.1 (+5.4)	79.5 (+6.0)	86.4 (+6.8)	82.64
√		√			82.5 (+1.8)	74.6 (+1.1)	80.7 (+1.1)	90.90
√			√		86.4 (+5.7)	76.3 (+2.8)	82.1 (+2.5)	100
√				√	86.1 (+5.4)	77.2 (+3.7)	81.7 (+2.1)	98.03
√	√	√	√	√	88.6 (+7.9)	83.2 (+9.7)	88.9 (+9.3)	87.71

In this table, √ means that the corresponding method was adopted.

**Table 3:** Comparison of STC-YOLO with state-of-the-art methods on the enhanced TT100K dataset.

Method	Size (Pixels)	AP (%)	AR (%)	mAP (%)	Params (M)	Speed (FPS)
YOLOv3	640 × 640	83.5	77	83.1	61.8	36.1
YOLOv6	640 × 640	81.7	69	81.3	18.5	19.92
YOLOv7	640 × 640	62.2	52.8	57.1	37.4	26.73
YOLOv5s	640 × 640	80.7	73.5	79.6	7.1	101.01
STC-YOLO	640 × 640	88.6	83.2	88.9	6.7	87.71

**Table 4:** Detailed mAP values of each class on the enhanced TT100K dataset (%).

Method	Total	i2	i4	i5	il100	il60	il80	io	ip	p10	p11	p12	p19	p23
YOLOv3	83.1	76.4	90.1	94.9	91.1	**99.5**	97.7	81	**87.3**	81.2	82.8	69.2	78.1	81
YOLOv6	81.3	80.2	83.4	92.6	**99.2**	97.2	96.3	82	87.2	76.1	79.3	71.2	77.8	78.1
YOLOv7	57.1	68.9	76.9	86.8	97.8	89.9	83.9	83.8	62.8	35.9	74.6	60	27.6	62.5
YOLOv5s	79.6	81	88.4	94.7	94.2	99.4	**98.3**	79.4	83.3	79.9	72.1	70.4	77.9	75.6
STC-YOLO	**88.9**	**88.1**	**91.7**	**97.6**	96.9	99.1	98.1	**87.1**	83.1	**88.9**	**91.3**	**90**	**88.8**	**90.9**
Method	p27	p3	p5	p6	pg	ph4	ph4.5	ph5	pl100	pl120	pl20	pl30	pl40	pl50
YOLOv3	86.4	87.1	96.6	68.5	87.1	86.8	81.9	68.1	96.7	83.9	78.2	74.9	81.7	84.9
YOLOv6	86.9	76.2	82	87.2	**87.5**	85.4	73	67.9	94.5	**91.5**	65.5	70.7	73	71.2
YOLOv7	73.3	70.3	72	18.9	86.6	40.5	65.8	1.16	87.1	61.1	40.3	31.8	50.3	45.3
YOLOv5s	86.5	70.9	96.1	74.6	84.3	88.8	69.2	67	94.3	79.2	65.7	70.4	70.6	69.7
STC-YOLO	**91.7**	**92.4**	**99**	**89.9**	84.2	**94.1**	**83.2**	**77.1**	**98**	90.3	**82.8**	**81.2**	**90.5**	**90.5**
Method	pl80	pm20	pm30	pm55	pn	pne	po	pr40	p26	pl5	pl60	pl70	w57	w59
YOLOv3	85.5	75.4	51.1	85.8	93.3	95.6	76.9	96.4	91.8	71.1	84	81.9	87.6	93.7
YOLOv6	72.3	**79.9**	42.7	87.5	90.6	92.8	71.8	96.9	82.4	**75.7**	70.2	**89.6**	90.5	**99.8**
YOLOv7	48.9	23.4	28.8	50.7	**95.1**	93.7	52.4	41.4	71.6	57.8	34	53.1	46.7	71.7
YOLOv5s	73.8	78.8	62	76.9	91.1	94	69.9	97.3	82.8	68.3	68.7	74.5	87.1	97.3
STC-YOLO	**89.1**	72.9	**75**	**88.5**	94.6	**95.9**	**81.8**	**98.4**	**93.2**	75.2	**88.7**	85.6	**93.2**	93.4
Method	wo	w13	w32	w55										
YOLOv3	60.8	90.2	75.3	68.9										
YOLOv6	54.2	89.6	74.6	84										
YOLOv7	13.1	41.9	16	65.3										
YOLOv5s	57.1	83.2	63.8	71.9										
STC-YOLO	**74.9**	**95.7**	**84.5**	**86.7**										

All results were obtained using the same hardware. In this table, the best results are in bold.

**Table 5:** Comparison of STC-YOLO with state-of-the-art methods on the original TT100K dataset.

Method	Backbone	Size (Pixels)	mAP (%)	Speed (FPS)	GPU
SSD+AlignedMatching [46]	VGG16	1024 × 1024	84.7	-	3090
TSR-SA [47]	CSPDarknet53	608 × 608	89.9	58.1	V100
PSG-Yolov5 [48]	CSPDarknet53	640 × 640	89.2	85.5	V100
AIE-YOLO [21]	CSPDarknet53	640 × 640	84.8	100.7	3090
YOLOv5s	CSPDarknet53	640 × 640	77	97.08	3090
STC-YOLO	CSPDarknet53	640 × 640	89.9	88.49	3090

**Table 6:** Comparison of STC-YOLO with state-of-the-art methods on the CCTSDB2021 dataset.

Method	Backbone	Size (Pixels)	mAP (%)	Speed (FPS)	GPU
ESSD [37]	VGG16	512 × 512	90.52	25.08	2080
YOLOv3 + MAF + SIA [49]	Darknet53	640 × 640	86.1	178.57	2080Ti
M-YOLO [38]	MobileNetv3	-	97.8	84	2080Ti
Faster R-CNN + ACFPN + Auto Augment [50]	ResNet50-D	-	99.5	29.8	V100
YOLOv5s	CSPDarknet53	640 × 640	98.2	81.3	3090
STC-YOLO	CSPDarknet53	640 × 640	99.2	81.96	3090

## Data Availability

The data that support the findings of this study are available upon request from the authors.

## Supplementary Materials

The following supporting information can be downloaded at: https://www.mdpi.com/article/10.3390/s23115307/s1, Table S1: Detailed mAP values of each class on the VisDrone2019 dataset. References [51,52,53,54,55,56] are cited in the supplementary materials.

## Abbreviations

TT100K	Tsinghua-Tencent 100K
STC-YOLO	Small traffic sign detection network for complex environments based on YOLO
CNN	Convolutional neural network
NWD	Normalized Gaussian Wasserstein distance
IoU	Intersection over union
CCTSDB	CSUST Chinese Traffic Sign Detection Benchmark
R-CNN	Region-based convolution neural network
YOLO	You only look once
SSD	Single shot multibox detector
mAP	Mean average precision
FPS	Frames per second
PFM	Receptive field module
PAN	Path aggregation network
GTSDB	German Traffic Sign Detection Benchmark
SPIE	International Society of Optics and Photonics
FPN	Feature pyramid network
LISA	Intelligent and Safe Automobiles
MPANet	Multiscale path aggregation network
C4STB	Four convolution modules and Swin Transformer Block
STB	Swin Transformer Block
W-MSA	Windows multi-head self-attention
SW-MSA	Shift windows multi-head self-attention
MLP	Multi-layer perceptron
LN	LayerNorm
MSA	Multi-head self-attention
SGD	Stochastic gradient descent

## References

[1] Zhang T., Zou J., Jia W. (2018). Fast and robust road sign detection in driver assistance systems. Appl. Intell..

[2] Wang C.W., You W.H. (2013). Boosting-SVM: Effective learning with reduced data dimension. Appl. Intell..

[3] Souani C., Faiedh H., Besbes K. (2014). Efficient algorithm for automatic road sign recognition and its hardware implementation. J. Real-Time Image Process..

[4] Yu L., Xia X., Zhou K. (2019). Traffic sign detection based on visual co-saliency in complex scenes. Appl. Intell..

[5] Greenhalgh J., Mirmehdi M. (2012). Real-time detection and recognition of road traffic signs. IEEE Trans. Intell. Transp. Syst..

[6] Berkaya S.K., Gunduz H., Ozsen O., Akinlar C., Gunal S. (2016). On circular traffic sign detection and recognition. Expert Syst. Appl..

[7] Ren S., He K., Girshick R., Sun J. (2015). Faster r-cnn: Towards real-time object detection with region proposal networks. IEEE Trans. Pattern Anal. Mach. Intell..

[8] Cai Z., Vasconcelos N. Cascade r-cnn: Delving into high quality object detection. Proceedings of the IEEE Conference on Computer Vision and Pattern Recognition.

[9] He K., Gkioxari G., Dollár P., Girshick R. Mask r-cnn. Proceedings of the IEEE International Conference on Computer Vision.

[10] Redmon J., Farhadi A. (2018). Yolov3: An incremental improvement. arXiv.

[11] Li C., Li L., Jiang H., Weng K., Geng Y., Li L., Ke Z., Li Q., Cheng M., Nie W. (2022). YOLOv6: A single-stage object detection framework for industrial applications. arXiv.

[12] Wang C.Y., Bochkovskiy A., Liao H.Y.M. (2022). YOLOv7: Trainable bag-of-freebies sets new state-of-the-art for real-time object detectors. arXiv.

[13] Liu W., Anguelov D., Erhan D., Szegedy C., Reed S., Fu C.Y., Berg A.C. (2016). Ssd: Single shot multibox detector. Proceedings of the Computer Vision–ECCV 2016: 14th European Conference.

[14] Fu C.Y., Liu W., Ranga A., Tyagi A., Berg A.C. (2017). Dssd: Deconvolutional single shot detector. arXiv.

[15] Zhang H., Qin L., Li J., Guo Y., Zhou Y., Zhang J., Xu Z. (2020). Real-time detection method for small traffic signs based on Yolov3. IEEE Access.

[16] Zhu Z., Liang D., Zhang S., Huang X., Li B., Hu S. Traffic-sign detection and classification in the wild. Proceedings of the IEEE Conference on Computer Vision and Pattern Recognition.

[17] Wu J., Liao S. (2022). Traffic sign detection based on SSD combined with receptive field module and path aggregation network. Comput. Intell. Neurosci..

[18] Liu S., Qi L., Qin H., Shi J., Jia J. Path aggregation network for instance segmentation. Proceedings of the IEEE Conference on Computer Vision and Pattern Recognition.

[19] Houben S., Stallkamp J., Salmen J., Schlipsing M., Igel C. (2013). Detection of traffic signs in real-world images: The German Traffic Sign Detection Benchmark. In Proceedings of the 2013 International Joint Conference on Neural Networks (IJCNN).

[20] Zhang J., Zou X., Kuang L.D., Wang J., Sherratt R.S., Yu X. (2022). CCTSDB 2021: A more comprehensive traffic sign detection benchmark. Human-Centric Computing and Information Sciences.

[21] Yan B., Li J., Yang Z., Zhang X., Hao X. (2022). AIE-YOLO: Auxiliary Information Enhanced YOLO for Small Object Detection. Sensors.

[22] Hnewa M., Radha H. (2021). Multiscale domain adaptive yolo for cross-domain object detection. Proceedings of the 2021 IEEE International Conference on Image Processing (ICIP).

[23] Fan B.B., Yang H. (2021). Multi-scale traffic sign detection model with attention. Proc. Inst. Mech. Eng. Part D J. Automob. Eng..

[24] Zhou K., Zhan Y., Fu D. (2021). Learning region-based attention network for traffic sign recognition. Sensors.

[25] Zhaosheng Y., Tao L., Tianle Y., Chengxin J., Chengming S. (2022). Rapid Detection of Wheat Ears in Orthophotos From Unmanned Aerial Vehicles in Fields Based on YOLOX. Front. Plant Sci..

[26] Bai Y., Zhang Y., Ding M., Ghanem B. Sod-mtgan: Small object detection via multi-task generative adversarial network. Proceedings of the European Conference on Computer Vision (ECCV).

[27] Bai Y., Zhang Y., Ding M., Ghanem B. Finding tiny faces in the wild with generative adversarial network. Proceedings of the IEEE Conference on Computer Vision and Pattern Recognition.

[28] Liu S., Huang D. Receptive field block net for accurate and fast object detection. Proceedings of the European Conference on Computer Vision (ECCV).

[29] Yu F., Koltun V., Funkhouser T. Dilated residual networks. Proceedings of the IEEE Conference on Computer Vision and Pattern Recognition.

[30] Lin T.Y., Dollár P., Girshick R., He K., Hariharan B., Belongie S. Feature pyramid networks for object detection. Proceedings of the IEEE Conference on Computer Vision and Pattern Recognition.

[31] Gómez-Moreno H., Maldonado-Bascón S., Gil-Jiménez P., Lafuente-Arroyo S. (2010). Goal evaluation of segmentation algorithms for traffic sign recognition. IEEE Trans. Intell. Transp. Syst..

[32] Salti S., Petrelli A., Tombari F., Fioraio N., Di Stefano L. (2015). Traffic sign detection via interest region extraction. Pattern Recognit..

[33] Fang C.Y., Chen S.W., Fuh C.S. (2003). Road-sign detection and tracking. IEEE Trans. Veh. Technol..

[34] Barnes N., Zelinsky A., Fletcher L.S. (2008). Real-time speed sign detection using the radial symmetry detector. IEEE Trans. Intell. Transp. Syst..

[35] Møgelmose A., Liu D., Trivedi M.M. (2015). Detection of US traffic signs. IEEE Trans. Intell. Transp. Syst..

[36] Zhang J., Xie Z., Sun J., Zou X., Wang J. (2020). A cascaded R-CNN with multiscale attention and imbalanced samples for traffic sign detection. IEEE Access.

[37] Sun C., Wen M., Zhang K., Meng P., Cui R. (2021). Traffic sign detection algorithm based on feature expression enhancement. Multimed. Tools Appl..

[38] Liu Y., Shi G., Li Y., Zhao Z. (2022). M-YOLO: Traffic sign detection algorithm applicable to complex scenarios. Symmetry.

[39] Liu Z., Lin Y., Cao Y., Hu H., Wei Y., Zhang Z., Lin S., Guo B. Swin transformer: Hierarchical vision transformer using shifted windows. Proceedings of the IEEE/CVF International Conference on Computer Vision.

[40] Zheng Z., Wang P., Liu W., Li J., Ye R., Ren D. Distance-IoU loss: Faster and better learning for bounding box regression. Proceedings of the AAAI Conference on Artificial Intelligence.

[41] Zhang Y.F., Ren W., Zhang Z., Jia Z., Wang L., Tan T. (2022). Focal and efficient IOU loss for accurate bounding box regression. Neurocomputing.

[42] Gevorgyan Z. (2022). SIoU loss: More powerful learning for bounding box regression. arXiv.

[43] Wang J., Xu C., Yang W., Yu L. (2021). A normalized Gaussian Wasserstein distance for tiny object detection. arXiv.

[44] Yu Z., Huang H., Chen W., Su Y., Liu Y., Wang X. (2022). YOLO-FaceV2: A Scale and Occlusion Aware Face Detector. arXiv.

[45] Lin T.Y., Maire M., Belongie S., Bourdev L., Girshick R., Hays J., Perona P., Ramanan D., Zitnick C.L., Dollar P. (2014). Microsoft coco: Common objects in context. Proceedings of the Computer Vision–ECCV 2014, 13th European Conference.

[46] Kang S.H., Park J.S. (2023). Aligned Matching: Improving Small Object Detection in SSD. Sensors.

[47] Chen J., Jia K., Chen W., Lv Z., Zhang R. (2022). A real-time and high-precision method for small traffic-signs recognition. Neural Comput. Appl..

[48] Hu J., Wang Z., Chang M., Xie L., Xu W., Chen N. (2022). PSG-Yolov5: A Paradigm for Traffic Sign Detection and Recognition Algorithm Based on Deep Learning. Symmetry.

[49] Zhang J., Ye Z., Jin X., Wang J., Zhang J. (2022). Real-time traffic sign detection based on multiscale attention and spatial information aggregator. J. Real-Time Image Process..

[50] Li X., Xie Z., Deng X., Wu Y., Pi Y. (2022). Traffic sign detection based on improved faster R-CNN for autonomous driving. J. Supercomput..

[51] Du D., Zhu P., Wen L., Bian X., Ling H., Hu Q., Peng T., Zheng J., Wang X., Zhang Y. VisDrone-DET2019: The vision meets drone object detection in image challenge results. Proceedings of the IEEE/CVF International Conference on Computer Vision Workshops.

[52] Liao J., Piao Y., Su J., Cai G., Huang X., Chen L., Huang Z., Wu Y. (2021). Unsupervised Cluster Guided Object Detection in Aerial Images. IEEE J. Sel. Top. Appl. Earth Obs. Remote Sens..

[53] Sun W., Dai L., Zhang X., Chang P., He X. (2022). RSOD: Real-time small object detection algorithm in UAV-based traffic monitoring. Appl. Intell..

[54] Liu B., Luo H., Wang H., Wang S. (2022). YOLOv3_ReSAM: A small-target detection method. Electronics.

[55] Zhu X., Lyu S., Wang X., Zhao Q. TPH-YOLOv5: Improved YOLOv5 based on transformer prediction head for object detection on drone-captured scenarios. Proceedings of the IEEE/CVF International Conference on Computer Vision.

[56] Liao J., Liu Y., Piao Y., Su J., Cai G., Wu Y. (2022). GLE-Net: A global and local ensemble network for aerial object detection. Int. J. Comput. Intell. Syst..

