# Supplementary material for: STC-YOLO: Small Object Detection Network for Traffic Signs in Complex Environments
Source: Sensors (Basel). 2023 Jun 3;23(11):5307. doi: 10.3390/s23115307 (PMC10255978; doi:10.3390/s23115307)
Supplement: Supplementary file 1 [file sensors-23-05307-s001.zip › sensors-2413664-supplementary.pdf]

STC-YOLO: Small Object Detection Network for Traffic Signs in Complex Environments

Huaqing Lai, Liangyan Chen *, Weihua Liu, Zi Yan and Sheng Ye

School of Electric and Electronic Engineering, Wuhan Polytechnic University, Wuhan 430023, China; lhq15527756213@163.com (H. L.); whliu2020@whpu.edu.cn (W. L.); yz9915@163.com (Z. Y.); yesheng19982023@163.com (S. Y.);
*Correspondence: chenliangyan@whpu.edu.cn; Tel.: +86 153-4260-4619.

1. Experimental Results and Analysis

1.1 Experimental Dataset

The VisDrone2019 dataset [**Error! Reference source not found.**] was gathered by the AISKYEYE team at Tianjin University's machine learning and data mining lab. The benchmark dataset provides 288 video clips with a total of 10209 still images captured by various drone cameras (6471 for training, 3190 for testing, and 548 for validation). The dataset has 10 object categories: (pedestrian, people, bicycle, car, van, truck, tricycle, awning-tricycle, bus, and motor). The dataset covers different weather, and lighting conditions, containing many small objects with different degrees of dense object deformation and occlusion.

1.2 Performance on the VisDrone2019 dataset

The comparative experiments on the proposed method and other object detection algorithms on the VisDrone2019 dataset was conducted to verify the average precision (AP) of different methods for UAV aerial objects, and the results are shown in Table S1. It can be seen from Table S1 that STC-YOLO outperformed YOLOv5 in detecting pedestrians, people, bicycle, car, van, truck, tricycle, awning-tricycle, bus, and motor in terms of AP. Specifically, this model obtained 10.2%, 10%, 9.9%, 5.7%, and 19.9% performance gains in terms of AP over 10 object categories, compared with the UCGNet [**Error! Reference source not found.**], RSOD [**Error! Reference source not found.**], YOLOv3_ReSAM [**Error! Reference source not found.**], TPH_YOLOv5 [**Error! Reference source not found.**], and GLE-Net [**Error! Reference source not found.**], respectively. Compared to YOLOv5, STC-YOLO improved the mAP by 7% (from 36% to 43%).

Table S1. Detailed mAP values of each class on the on the VisDrone2019 dataset (%).

Method	All	pedestrian	people	bicycle	car	van	truck	tricycle	awning-tricycle	bus	motor
UCGNet [**Error! Reference source not found.**]	32.8	34	22.8	19.4	58.6	39.6	33	23.1	14.5	52.4	30.9
RSOD [**Error! Reference source not found.**]	33	29.9	24	9.38	72.1	40	31.6	24.4	13.5	51.2	34.2
YOLOv3_ReSAM [**Error! Reference source not found.**]	33.1	17.28	15.78	**28.7**	**84.3**	**53.7**	42.9	30.4	24.3	**62.3**	**61.5**
TPH_YOLOv5 [**Error! Reference source not found.**]	37.3	29	16.75	15.69	68.9	49.7	**45.1**	27.3	**24.7**	61.8	30.9

GLE-Net [**Error! Reference source not found.**]	23.1	19.2	12.4	6.9	53.1	28.8	26.1	15.2	10	41.3	18.4
YOLOv5	36	37	29.8	12.8	75.4	38.7	35.7	25	12.3	54	38.8
STC-YOLO	**43**	**48.6**	**41.4**	21.6	79.9	46.9	40.2	**31.5**	20.1	56.3	43.8

All results were obtained using the same hardware. In this table, the best results are in bold.

References

1. Du, D.; Zhu, P.; Wen, L.; Bian, X.; Ling, H.; Hu, Q.; Peng, T.; Zheng, J.; Wang, X.; Zhang, Y.; et al. VisDrone-DET2019: The vision meets drone object detection in image challenge results. In Proceedings of the IEEE/CVF International Conference on Computer Vision Workshops, Seoul, Korea, 27–28 October 2019.
2. Liao, J.; Piao, Y.; Su, J.; Cai, G.; Huang, X.; Chen, L.; Huang, Z.; Wu, Y. Unsupervised Cluster Guided Object Detection in Aerial Images. *IEEE J. Sel. Top. Appl. Earth Obs. Remote Sens.* **2021**, *14*: 11204–11216.
3. Sun, W.; Dai, L.; Zhang, X.; Chang, P.; He, X. RSOD: Real-time small object detection algorithm in UAV-based traffic monitoring. *Appl. Intell.* **2021**: 1–16.
4. Liu, B.; Luo, H.; Wang, H.; Wang, S. YOLOv3_ReSAM: A small-target detection method. *Electron.*, **2022**, *11(10)*: 1635.
5. Zhu, X.; Lyu, S.; Wang, X.; Zhao, Q. TPH-YOLOv5: Improved YOLOv5 based on transformer prediction head for object detection on drone-captured scenarios. In Proceedings of the IEEE/CVF international conference on computer vision, Montreal, QC, Canada, 10-17 October 2021; pp: 2778–2788.
6. Liao, J.; Liu, Y.; Piao, Y.; Su, J.; Cai, G.; Wu, Y. GLE-Net: A global and local ensemble network for aerial object detection. *Int. J. Comput. Intell. Syst.* **2022**, *15(1)*: 2.
